# Spectrum and immunovirological determinants of tumours among people living with HIV in Uganda: A retrospective cross sectional study from a specialised HIV centre, 2017–2026

**DOI:** 10.1371/journal.pone.0358066

**Published:** 2026-09-22

**Authors:** Joseph Mwaka, Conrad Sserunjogi, Jesca Natalayi

**Affiliations:** Mildmay Research Centre, Kampala, Uganda; Gulu University, UGANDA

## Abstract

**Background:**

Sub-Saharan Africa carries a disproportionate burden of HIV-associated malignancies. The contemporary tumour spectrum in the dolutegravir (DTG) era remains incompletely characterised in East African routine HIV-care settings. The immunovirological context at the time of cancer or benign tumor diagnosis is similarly under-described. We sought to describe the spectrum, malignant fraction, and immunovirological correlates of neoplasms diagnosed at a specialised HIV centre in Kampala, Uganda.

**Methods:**

We conducted a retrospective, cross-sectional analysis of 219 tumour records identified within the longitudinal clinical cohort of people living with HIV (PLHIV) in continuous care at Mildmay Hospital, contributed by 216 individual patients (three of whom each had two separate tumour diagnoses on different dates) between November 2017 and January 2026. Tumours were classified by ICD-10 disease group and malignancy status (benign/malignant). Bivariate associations with malignancy status were tested using χ² or Fisher exact tests for categorical variables and Mann–Whitney U tests for continuous variables (two-sided α = 0.05), with Benjamini–Hochberg false discovery rate (FDR) correction applied across each panel of comparisons given the number of variables tested. Crude odds ratios (OR) with 95% confidence intervals (CI) were computed for dichotomous comparisons. A sensitivity analysis restricted to one tumour record per patient (n = 216) was performed to assess the influence of the three patients with two records each.

**Results:**

The cohort had a median age of 47 years (IQR 39–54) and was 127/219 (58.0%) female. Overall, 138/219 tumours (63.0%) were malignant. Kaposi sarcoma (KS) was the most frequently registered malignancy (n = 65; 47.1% of all malignant tumours; 29.7% of the full cohort). Among 155 records with viral load data, 133 (85.8%) were virally suppressed at tumour diagnosis, yet 70/133 (52.6%) of those suppressed records were malignant. Advanced WHO clinical stage (III/IV) at ART initiation was associated with malignant diagnosis (OR 3.51, 95% CI 1.54–8.81; FDR-adjusted p = 0.003). Median CD4 at ART initiation was lower among malignant compared with benign tumour records (147 vs 476 cells/µL; FDR-adjusted p = 0.001), and median ART duration before diagnosis was shorter (37 vs 101 months; FDR-adjusted p < 0.001). Restricting the analysis to KS versus other malignancies only (n = 138), KS patients had lower CD4 at ART initiation (136 vs 171 cells/µL, p = 0.079) and markedly shorter ART duration before diagnosis (4 vs 63 months, p < 0.001) than patients with other malignancies. All associations were materially unchanged in the patient-level sensitivity analysis.

**Conclusions:**

HIV-associated tumours in this Ugandan cohort remain predominantly malignant and are dominated by KS despite high viral suppression rates. Malignancy clusters among patients with low pre-ART CD4 counts, advanced WHO stage, and short ART duration, though these are unadjusted, hypothesis-generating comparisons rather than evidence of causal or temporal gradients. These data establish the analytic platform for a prospective HIV-oncology cohort at Mildmay Hospital and Mildmay Research Centre and inform context-appropriate cancer screening priorities.

## 1. Introduction

The relationship between HIV infection and cancer has been fundamentally reshaped by the global scale-up of combination antiretroviral therapy (ART). In the pre-ART era, three AIDS-defining cancers, Kaposi sarcoma (KS), non-Hodgkin lymphoma (NHL), and invasive cervical cancer, dominated the malignancy landscape of people living with HIV (PLHIV). These cancers were direct manifestations of advanced immunosuppression [1,2]. Sustained viral suppression has since changed this picture in high-income settings: the relative *incidence* of AIDS-defining cancers has declined, while the incidence of non-AIDS-defining cancers (NADCs), including hepatocellular, lung, anal, and colorectal carcinomas, has risen [3–6]. This rise is attributed to an ageing PLHIV population, chronic inflammation, and persistent oncogenic co-infections such as human papillomavirus (HPV), hepatitis B and C viruses, Epstein–Barr virus (EBV), and human herpesvirus-8 (HHV-8).

In sub-Saharan Africa, this epidemiological transition is incomplete and geographically uneven. Among registered malignancies in PLHIV across East Africa, KS is consistently the single most frequently reported cancer type, a pattern attributed to regional HHV-8 seroprevalence exceeding 40% in some adult population studies and to persistent immune reconstitution gaps following late ART initiation [7–10]. Uganda’s Kampala Cancer Registry, Africa’s longest-running population-based cancer registry, has recorded an age-standardised KS incidence rate of approximately 29.6 per 100,000 population in its most recent observed period, among the highest such rates reported for any population-based registry, though on a declining trajectory as ART coverage has expanded [11–13]. National data from this registry document a continuing predominance of infection-associated malignancies among PLHIV, even in the contemporary treatment era [14–16].

Uganda adopted dolutegravir (DTG)-based ART as its preferred first-line regimen in 2018, with phased national roll-out beginning that year following WHO’s 2018–2019 guidance [17–19]. Despite this policy transition, the cancer spectrum among Ugandan PLHIV in the DTG era has not been systematically characterised within specialised HIV clinical settings. Most published evidence derives either from population-based cancer registries that lack ART history and virological data, or from oncology units that under-record HIV exposure [14,19,20]. Consequently, the joint immunological-virological-oncological profile of tumours diagnosed in routine HIV care, including the contribution of co-occurring benign neoplasms, remains poorly defined.

Three specific evidence gaps motivate this study. First, the proportion of tumours that are malignant versus benign in HIV clinic populations is rarely reported, yet it is operationally important: benign tumours such as uterine leiomyomas and benign prostatic hyperplasia consume substantial diagnostic and surgical capacity and may be modulated by chronic ART exposure [21,22]. Second, the immunovirological context at cancer diagnosis, viral load, CD4 at ART initiation, WHO stage at ART initiation, ART duration, and ART regimen class, is needed to identify subgroups at greatest residual risk in an era of high population-level viral suppression. Third, with the growing push towards integrated HIV–non-communicable disease (NCD) services, data anchored in HIV clinical settings are needed to inform context-appropriate cancer screening and referral pathways [23,24].

Mildmay Hospital is a specialised HIV hospital with an embedded research arm, Mildmay Research Centre, in Lweza, Kampala, Uganda, with longitudinal electronic records spanning the policy transition from efavirenz (EFV)-based to DTG-based first-line ART. We used nearly a decade of these records to provide an immunovirologically contextualised description of tumours diagnosed among PLHIV at a Ugandan specialised HIV centre. The findings are intended to establish the epidemiological foundation for a prospective HIV-oncology cohort and to directly inform site-level surveillance, diagnostic, and referral priorities.

## 2. Methods

### 2.1 Study design and setting

This study draws on tumour records identified retrospectively within the longitudinal clinical cohort of PLHIV in continuous care at Mildmay Hospital, Kampala, Uganda. Because a complete denominator of all PLHIV in care and the corresponding person-time at risk was not available, incidence rates could not be calculated. The comparative analyses presented here (malignant versus benign tumour characteristics) are therefore cross-sectional in design: they evaluate characteristics recorded at the time of ART initiation and tumour diagnosis rather than following individual patients prospectively over time. Mildmay is a specialised HIV centre providing comprehensive HIV testing, ART initiation, virological monitoring, and management of HIV-related comorbidities, including referral pathways for malignancies. Mildmay Research Centre, the research arm of Mildmay Uganda, is co-located with Mildmay Hospital. The centre’s electronic data system has captured ICD-10-coded diagnoses since 2017. There are approximately 14,500 patients in active care and over 17,000 patient records (as of January 2026).

### 2.2 Study population and case definition

All PLHIV with at least one ICD-10 tumour diagnosis recorded in the hospital’s electronic medical registry between 1 November 2017 and 29 January 2026 were eligible. Data were accessed for research purposes on 13 March 2026. The primary analytical unit was the tumour record, irrespective of the modality by which it was diagnosed. Diagnoses were established through routine clinical care using the modality appropriate to the presenting tumour (for example, histopathology for accessible lesions such as cutaneous KS or cervical biopsies, and clinical or radiological criteria for others, such as hepatocellular carcinoma or CNS tumours); the specific diagnostic modality was not uniformly recorded across tumour types and could not be systematically compared, which we address as a limitation. Applying this case definition identified 219 tumour records contributed by 216 individual patients; three patients contributed two distinct primary tumour records each, on different dates. This is disclosed here and addressed in the Limitations. One further record, in which WHO clinical stage was coded as an undefined “unknown” category outside the four-stage WHO scale, was excluded from the analytic dataset entirely, as this value could not be assigned a valid stage or treated as missing-at-random within the ordinal WHO stage variable; all descriptive and inferential statistics reported below reflect this final analytic dataset of 219 records. Tumours were classified as malignant or benign based on ICD-10 coding and aggregated into five clinically meaningful groups: Kaposi sarcoma, haematopoietic malignancies, gynaecological tumours, prostate-related tumours, and other solid tumours.

### 2.3 Variables and definitions

Demographic variables: age at tumour diagnosis (years; categorised <30, 30–44, 45–59, ≥ 60 for descriptive presentation) and sex. Immunological variables: CD4 cell count at ART initiation (cells/µL; categorised <200, 200–499, ≥ 500) and WHO clinical stage at ART initiation (I, II, III, IV; the World Health Organization’s four-stage clinical staging system for HIV disease severity). Virological variables: plasma HIV RNA at tumour diagnosis, dichotomised as suppressed (<1,000 copies/mL) or unsuppressed (≥1,000 copies/mL) per the Uganda Ministry of Health threshold in use during the study period [25]. Treatment variables: ART backbone at tumour diagnosis, ART regimen category (DTG-based; EFV-based; other, predominantly protease-inhibitor- or nevirapine-based), and ART duration from ART initiation to tumour diagnosis in months (categorised ≤12, 13–60, 61–120, > 120 months for descriptive presentation). Continuous variables were categorised into these clinically standard bands for descriptive Table presentation only; all inferential comparisons of continuous variables used the untransformed continuous measure (see Section 2.5).

### 2.4 Data sources and quality

De-identified records were extracted from the Mildmay Hospital electronic clinical database for research purposes on 13 March 2026. ICD-10 codes were verified against the WHO ICD-10 reference [26]. Data quality checks included range validation for age, ART duration, and CD4 counts; cross-checks of WHO stage codes against textual entries, which identified and led to the exclusion of the single undefined-stage record described in Section 2.2; and reconciliation of duplicate patient identifiers. Missingness was substantial and variable-specific: CD4 at ART initiation was missing for 126/219 records (57.5%), viral load status for 64/219 (29.2%), ART regimen category for 42/219 (19.2%), WHO stage for 15/219 (6.8%), ART duration for 9/219 (4.1%), and age and sex for none. All analyses were performed on complete cases for each specific variable being tested; the denominator for every statistic reported in Results and in Tables 1, 3, and 4 is stated explicitly alongside that statistic. We consider it plausible that CD4 missingness is not missing completely at random, since patients transferring into care after ART initiation elsewhere, or those stable on long-term suppressive ART, are less likely to have a contemporaneous CD4 result on file; this is discussed further as a limitation.

**Table 1:** Baseline characteristics of the cohort (N = 219 tumour records from 216 individual patients).

Characteristic	n	%	Missing, n (%)
**Age, years, median (IQR)**	47	(IQR 39–54)	0 (0)
<30	14	6.4	
30–44	81	37.0	
45–59	89	40.6	
≥60	35	16.0	
**Sex**			0 (0)
Female	127	58.0	
Male	92	42.0	
**WHO clinical stage at ART initiation (n = 204)**			15 (6.8)
Stage I	101	49.5	
Stage II	55	27.0	
Stage III	23	11.3	
Stage IV	25	12.3	
**CD4 count at ART initiation, cells/µL, median (IQR) (n = 93)**	171	(IQR 87–423)	126 (57.5)
<200	52	55.9	
200–499	22	23.7	
≥500	19	20.4	
**ART duration before tumour diagnosis, months, median (IQR) (n = 210)**	61.5	(IQR 6–130)	9 (4.1)
≤12 months	62	29.5	
13–60 months	41	19.5	
61–120 months	44	21.0	
>120 months	63	30.0	
**ART regimen category at tumour diagnosis (n = 177)**			42 (19.2)
DTG-based	77	43.5	
EFV-based	50	28.2	
Other (PI-based / NVP-based)	50	28.2	
**HIV RNA status at tumour diagnosis (n = 155)**			64 (29.2)
Suppressed (<1,000 copies/mL)	133	85.8	
Unsuppressed (≥1,000 copies/mL)	22	14.2	
**Tumour status**			0 (0)
Malignant	138	63.0	
Benign	81	37.0	

ART, antiretroviral therapy; CD4, cluster of differentiation 4; DTG, dolutegravir; EFV, efavirenz; IQR, interquartile range; NVP, nevirapine; PI, protease inhibitor; WHO, World Health Organization.

### 2.5 Statistical analysis

Continuous variables were summarised as median (interquartile range, IQR); categorical variables as frequencies and percentages. The Shapiro–Wilk test was used to assess normality: CD4 count and ART duration departed significantly from a normal distribution (both p < 0.001), supporting the use of non-parametric methods for these variables; age was closer to normally distributed (p = 0.39) but was analysed using the same non-parametric approach as the other continuous variables, for methodological consistency. Bivariate associations between candidate exposures and malignancy status were tested with Pearson χ² or Fisher exact tests (the latter used whenever any expected cell count was < 5) for categorical variables, and Mann–Whitney U tests for continuous variables. Crude odds ratios (OR, the ratio of the odds of malignancy in one group relative to a reference group) with 95% confidence intervals (CI) were computed for 2 × 2 comparisons using the conditional maximum-likelihood estimator. Because multiple bivariate comparisons were tested against the same outcome, we applied the Benjamini–Hochberg procedure to control the false discovery rate (FDR) within each panel of tests (the main malignancy panel and the KS-versus-other-malignancies panel were adjusted separately); both the nominal and FDR-adjusted p-values are reported. No multivariable regression was performed at this descriptive stage; all associations reported are unadjusted and hypothesis-generating rather than causal, and should not be interpreted as evidence of a dose-response or temporal gradient, since the data are cross-sectional and reflect a single tumour record per comparison rather than repeated measurement of the same patient over time.

The primary analytic unit was the tumour record (n = 219) rather than the patient, because the study’s focus is the spectrum and characteristics of tumours rather than patient-level incidence, and because only three of 216 patients (1.4%) contributed more than one record. To assess whether this choice materially affected conclusions, we repeated the full bivariate panel restricted to one tumour record per patient (the earliest, by date; n = 216) as a pre-specified sensitivity analysis; results are reported in the Results section and were materially unchanged. All analyses were conducted in R (version 4.4) using base statistics and the tidyverse framework.

### 2.6 Ethical considerations

The study used routinely collected, de-identified clinical data. Ethical approval was obtained from the Mildmay Uganda Research Ethics Committee (ref: MUREC-2025–808). Given the retrospective nature and use of de-identified records, the requirement for individual informed consent was waived. The study adhered to the Declaration of Helsinki [27] and STROBE reporting guidelines for observational studies [28].

## 3. Results

### 3.1 Cohort characteristics

A total of 219 tumour records from 216 individual patients met the inclusion criteria described in Section 2.2; three patients each contributed two distinct primary tumour records. The cohort had a median age of 47 years (IQR 39–54), and 127/219 (58.0%) were female. Most tumour records were registered between 2017 and 2019 (n = 129; 58.9%), with a peak in 2018 (n = 73) partly attributable to a centre-wide data quality improvement programme, followed by 10–19 new records per year thereafter through 2025 (Table 1); this temporal pattern in registration volume should not be interpreted as reflecting a true change in underlying cancer incidence, and is discussed further in the Limitations.

Missingness reflected routine data realities and varied substantially by variable: CD4 count at ART initiation was unavailable for 126/219 (57.5%) records, HIV RNA at tumour diagnosis was missing for 64/219 (29.2%), ART regimen category was unrecorded for 42/219 (19.2%), and WHO clinical stage was missing for 15/219 (6.8%). The high missingness for CD4 likely reflects that a substantial proportion of patients were enrolled in the electronic system only after initial laboratory values had been obtained elsewhere, and that patients stable on long-term suppressive ART are monitored less intensively; this pattern is plausibly not missing completely at random and is discussed as a limitation.

### 3.2 Tumour spectrum and malignant fraction

Of 219 tumours, 138 (63.0%) were malignant and 81 (37.0%) benign. Five disease groups accounted for the full cohort (Table 2). KS was the largest single disease group (n = 65, 29.7% of all tumours; 100% malignant), with cutaneous KS the dominant subtype (n = 47, 72.3%). Gynaecological tumours (n = 54, 24.7%) were predominantly benign uterine leiomyomas (n = 44, 81.5%), with 10 malignant cervical and other gynaecological cancers. The “other solid tumours” group (n = 53, 24.2%) was approximately evenly divided between benign (n = 25) and malignant (n = 28) tumours, encompassing hepatocellular carcinoma, brain tumours, colorectal, and skin cancers. Haematopoietic malignancies (n = 32, 14.6%; 100% malignant) were dominated by myelodysplastic syndromes (n = 10), lymphomas (n = 12, combined Hodgkin and non-Hodgkin), and multiple myeloma (n = 4). Prostate-related tumours (n = 15, 6.8%) were predominantly benign prostatic hyperplasia (n = 12), with three prostate cancers. Combined AIDS-defining cancers (KS, NHL, and invasive cervical cancer) represented 86/219 (39.3%) of the total cohort and 86/138 (62.3%) of all malignant tumours.

**Table 2:** Tumour spectrum by disease group and malignancy status, with leading ICD-10 diagnoses (N = 219).

Disease group / Leading ICD-10 diagnoses	Total n (%)	Malignant n (%)	Benign n (%)	Leading subtypes
**Kaposi sarcoma**	65 (29.7)	65 (100.0)	0 (0.0)	
Kaposi sarcoma of skin	47	47	—	HHV-8-associated; ICD-10: C46.0
Kaposi sarcoma of soft tissue	7	7	—	ICD-10: C46.1
Kaposi sarcoma of palate	6	6	—	ICD-10: C46.2
Kaposi sarcoma, other/unspecified	5	5	—	ICD-10: C46.7/C46.9
**Gynaecological tumours**	54 (24.7)	10 (18.5)	44 (81.5)	
Uterine leiomyoma (all subtypes)	44	—	44	ICD-10: D25.x; benign
Cervical malignancy (exo-/endocervix)	10	10	—	ICD-10: C53.x; AIDS-defining
**Other solid tumours**	53 (24.2)	28 (52.8)	25 (47.2)	
Benign lipomatous neoplasm (all sites)	12	—	12	ICD-10: D17.x
Squamous/basal cell carcinoma of skin	5	5	—	ICD-10: C44.x
Hepatic and gastrointestinal cancers	5	5	—	ICD-10: C22.x, C18–C20
Other (brain, breast, bladder, etc.)	31	18	13	Mixed NADCs
**Haematopoietic malignancies**	32 (14.6)	32 (100.0)	0 (0.0)	
Hodgkin and non-Hodgkin lymphoma	12	12	—	ICD-10: C81–C86; NHL AIDS-defining
Myelodysplastic syndromes/refractory anaemia	10	10	—	ICD-10: D46.x
Multiple myeloma	4	4	—	ICD-10: C90.0
Other (mycosis fungoides, leukaemia, polycythaemia)	6	6	—	ICD-10: C84, C91–C95, D45
**Prostate-related tumours**	15 (6.8)	3 (20.0)	12 (80.0)	
Benign prostatic neoplasm / hyperplasia	12	—	12	ICD-10: D29.1 / N40
Prostate cancer	3	3	—	ICD-10: C61; NADC
**TOTAL**	**219 (100)**	**138 (63.0)**	**81 (37.0)**	

ICD-10, International Classification of Diseases, 10th revision; NADC, non-AIDS-defining cancer; KS, Kaposi sarcoma; HHV-8, human herpesvirus-8; NHL, non-Hodgkin lymphoma.

### 3.3 Sex-stratified tumour pattern

The tumour pattern differed by sex. Among 127 women, gynaecological tumours were the largest single group (54/127, 42.5%), most of which were benign uterine leiomyomas (44/54, 81.5%). KS accounted for 24/127 (18.9%) of tumours in women. Among 92 men, KS was the largest group (41/92, 44.6%; all malignant), followed by other solid tumours (19/92, 20.7%) and haematopoietic malignancies (17/92, 18.5%). Overall, men had a higher malignant tumour fraction than women (73/92 [79.3%] vs 65/127 [51.2%]; χ² p < 0.001; crude OR for malignancy, male vs female, 3.64, 95% CI 1.91–7.16; FDR-adjusted p < 0.001). We discuss the likely drivers of this sex difference in Section 4.

### 3.4 Immunovirological profile and ART exposure at tumour diagnosis

**Fig 1:**
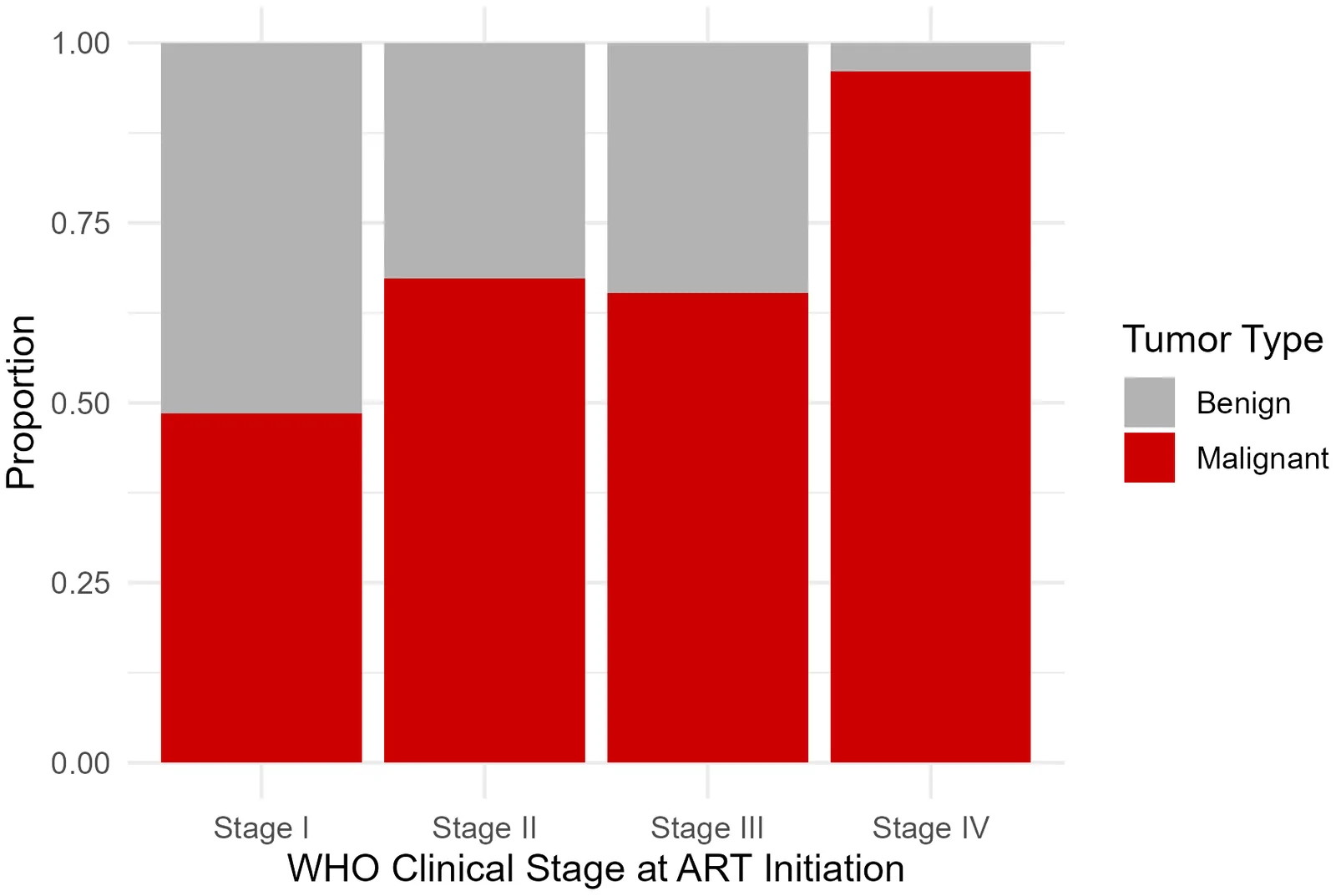
Proportion of malignant and benign tumours by WHO clinical stage at antiretroviral therapy (ART) initiation. Each bar represents 100% of tumour records within a given WHO clinical stage at ART initiation (Stage I through Stage IV; n = 204 records with non-missing WHO stage). Red indicates malignant tumours; grey indicates benign tumours. This is a cross-sectional comparison of proportions across stage categories, not a within-patient trend. The malignant proportion was 48.5% (49/101) at Stage I, rising to 96.0% (24/25) at Stage IV. Advanced WHO stage (III/IV) was associated with malignant diagnosis (crude OR 3.51, 95% CI 1.54–8.81; FDR-adjusted p = 0.003).

**Fig 2:**
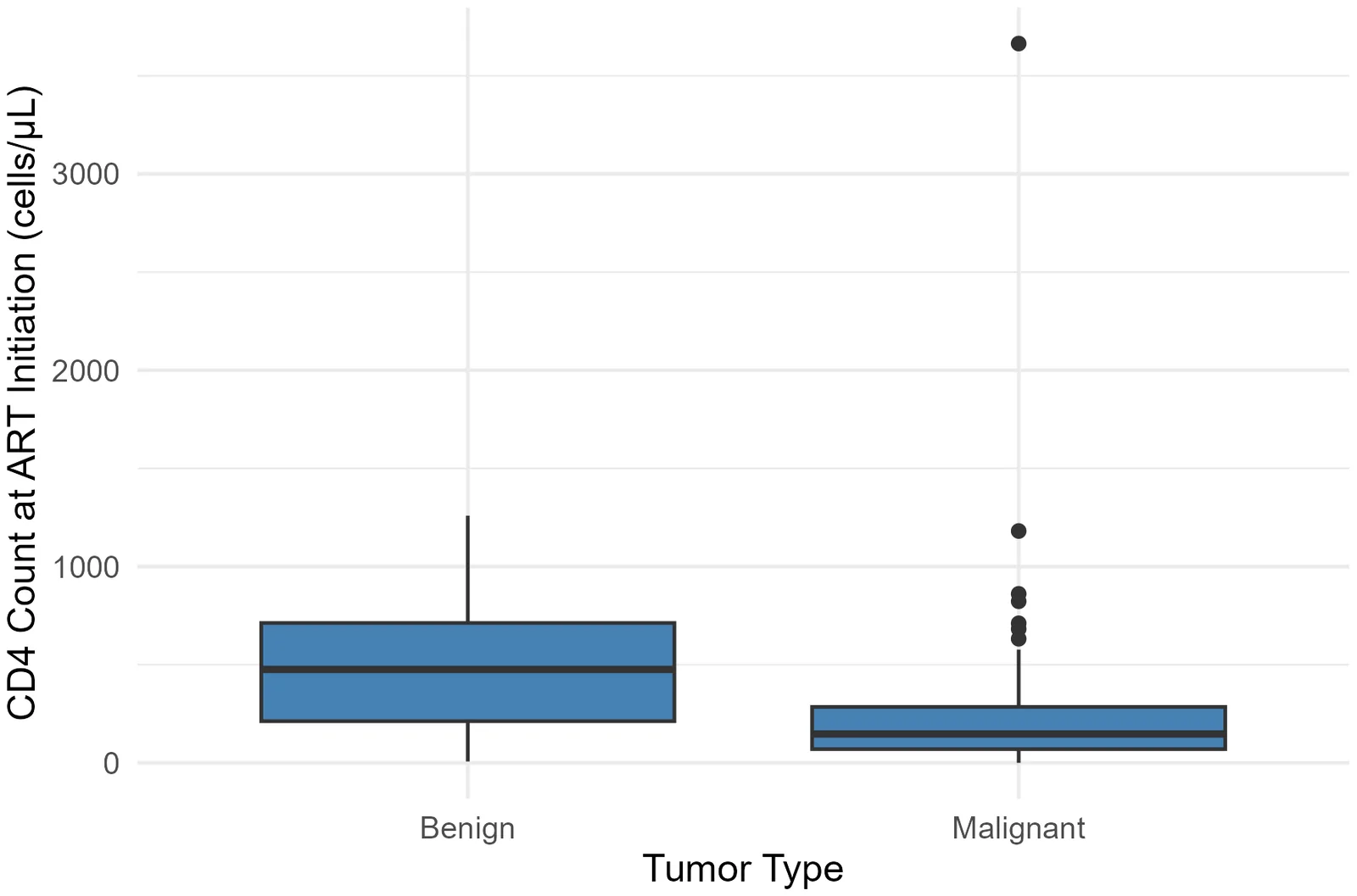
CD4 count at ART initiation by tumour type (benign vs. malignant). Box-and-whisker plots showing the distribution of CD4 T-cell counts (cells/µL) at ART initiation, stratified by tumour type. The horizontal line within each box is the median; box limits are the interquartile range (IQR); whiskers extend to 1.5 × IQR; points are outliers. Records subsequently associated with malignant tumours had a lower median CD4 count than those with benign tumours (147 vs. 476 cells/µL; Mann–Whitney U, FDR-adjusted p = 0.001). Data available for 93 of 219 records (57.5% missing).

**Fig 3:**
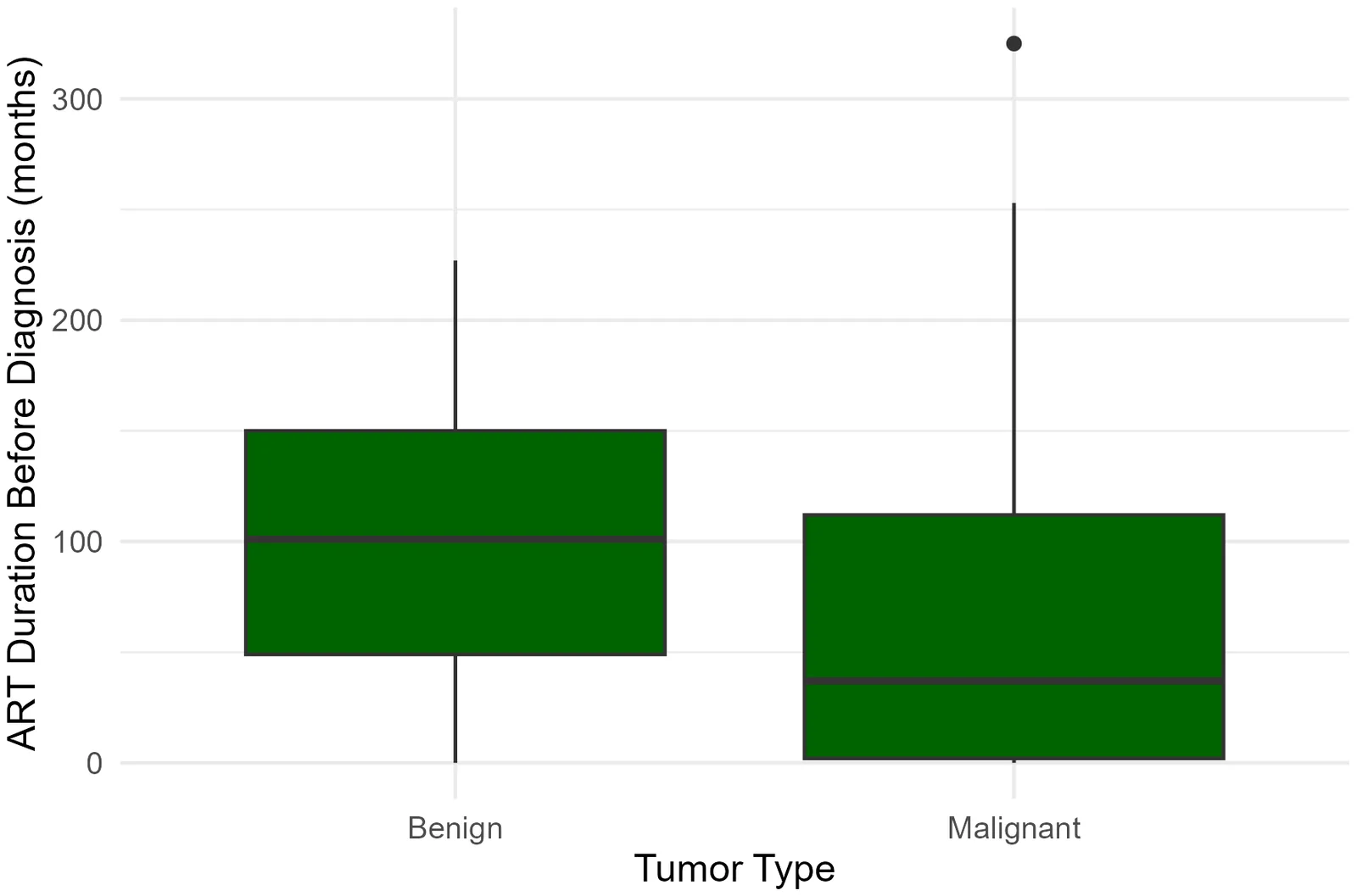
ART duration before tumour diagnosis by tumour type (benign vs. malignant). Box-and-whisker plots comparing ART duration (months) from ART initiation to tumour diagnosis, stratified by tumour type. Malignant tumour records had a shorter median ART duration before diagnosis (37 months; IQR 2–112) than benign tumour records (101 months; IQR 49–150; Mann–Whitney U, FDR-adjusted p < 0.001). Data available for 210 of 219 records (4.1% missing). This cross-sectional pattern is consistent with both IRIS-associated unmasking of subclinical malignancy and detection of pre-existing occult malignancy during early ART engagement (see Discussion); it should not be read as a longitudinal or causal gradient.

Bivariate associations with malignancy status are presented in Table 3 and illustrated in Figs 1–3. All p-values below are FDR-adjusted within this panel of ten comparisons unless stated otherwise; nominal (unadjusted) p-values are given in Table 3.

**Table 3:** Bivariate associations between patient and treatment characteristics and malignancy status (N = 219 tumour records).

Variable	Benign (n = 81)	Malignant (n = 138)	n analysed	Nominal p	FDR-adjusted p	OR (95% CI)
**Age, years, median (IQR)**	49 (42–54)	43 (37–54)	219	0.030	0.037	—
**Age category**			219	<0.001	0.001	—
<30	2 (2.5)	12 (8.7)				
30–44	22 (27.2)	59 (42.8)				
45–59	47 (58.0)	42 (30.4)				
≥60	10 (12.3)	25 (18.1)				
**Sex**			219	<0.001	<0.001	3.64 (1.91–7.16)
Female	62 (76.5)	65 (47.1)				Reference
Male	19 (23.5)	73 (52.9)				
**WHO clinical stage**			204	0.001	0.003	—
Stage I	52 (65.8)	49 (39.2)				
Stage II	18 (22.8)	37 (29.6)				
Stage III	8 (10.1)	15 (12.0)				
Stage IV	1 (1.3)	24 (19.2)				
Advanced (III/IV) vs Early (I/II)	9/79 (11.4)	39/86 (45.3)	204	0.002	0.003	3.51 (1.54–8.81)
**CD4 at ART initiation, cells/µL, median (IQR)**	476 (212–712)	147 (70–285)	93	<0.001	0.001	—
<200	5 (22.7)	47 (66.2)	93	<0.001	<0.001	—
200–499	6 (27.3)	16 (22.5)				
≥500	11 (50.0)	8 (11.3)				
**ART duration, months, median (IQR)**	101 (49–150)	37 (2–112)	210	<0.001	<0.001	—
≤12 months	8 (9.9)	54 (41.9)	210	<0.001	<0.001	—
13–60 months	20 (24.7)	21 (16.3)				
61–120 months	21 (25.9)	23 (17.8)				
>120 months	32 (39.5)	31 (24.0)				
**HIV RNA status**			155	0.127	0.141	2.39 (0.82–7.92)
Suppressed	63 (91.3)	70 (81.4)				Reference
Unsuppressed	6 (8.7)	16 (18.6)				
**ART regimen category**			177	0.981	0.981	—
DTG-based	28 (44.4)	49 (43.0)				
EFV-based	17 (27.0)	32 (28.1)				
Other (PI/NVP)	18 (28.6)	33 (28.9)				

*Tests: χ² for k × 2 contingency tables with all expected counts ≥5; Fisher exact otherwise; Mann–Whitney U for continuous variables. Percentages in parentheses are column percentages (denominator = records with non-missing data for that variable, stated in the “n analysed” column). p-values are FDR-adjusted (Benjamini–Hochberg) within this ten-comparison panel; nominal (unadjusted) p-values are also shown. OR, crude odds ratio; CI, confidence interval; DTG, dolutegravir; EFV, efavirenz; PI, protease inhibitor; NVP, nevirapine; IQR, interquartile range.*

#### WHO clinical stage at ART initiation.

Advanced WHO stage (III/IV) at ART initiation was associated with malignant tumour diagnosis: 39/86 (45.3%) of records with malignant tumours had advanced stage at ART start, compared with 9/79 (11.4%) of those with benign tumours (crude OR 3.51, 95% CI 1.54–8.81; FDR-adjusted p = 0.003). Among the 25 patients in WHO stage IV at ART initiation, 24 (96.0%) had a malignant tumour, of whom 20 (83.3%) had KS; at WHO stage I, 49/101 (48.5%) had a malignant tumour. This is a cross-sectional difference in proportions across WHO stage categories at a single point in time (illustrated as a stacked proportion chart in Fig 1), not a within-patient trend or longitudinal gradient, since no patient in this dataset was followed serially across stages.

#### CD4 count at ART initiation.

Among the 93 records with available CD4 data, median CD4 at ART initiation was lower in records with malignant tumours than those with benign tumours (147 vs 476 cells/µL; FDR-adjusted p = 0.001). In the categorical breakdown, 47/71 (66.2%) of records with pre-ART CD4 < 200 cells/µL had a malignant tumour, compared with 8/19 (42.1%) of those with CD4 ≥ 500 cells/µL (Fig 2). The high missingness for CD4 (57.5%) is discussed in Section 2.4 and in the Limitations.

#### ART duration before tumour diagnosis.

Median ART duration before tumour diagnosis was shorter for malignant tumours (37 months; IQR 2–112) than benign tumours (101 months; IQR 49–150; FDR-adjusted p < 0.001). Among records diagnosed within 12 months of ART initiation, 54/62 (87.1%) were malignant, compared with 31/64 (48.4%) among those on ART for >120 months (Fig 3). As with WHO stage, this is a cross-sectional comparison of proportions across categories of a single covariate, not evidence of a temporal or causal gradient; two non-mutually-exclusive explanations for this pattern, unmasking of subclinical malignancy by immune reconstitution inflammatory syndrome (IRIS), and detection of pre-existing occult malignancy during the intensified clinical contact that accompanies ART initiation, are considered in the Discussion.

#### HIV RNA at tumour diagnosis.

Among 155 records with viral load data, 133 (85.8%) were virally suppressed. A higher proportion of unsuppressed records were malignant than suppressed records (16/22, 72.7% vs 70/133, 52.6%; crude OR 2.39, 95% CI 0.82–7.92), but this difference did not reach statistical significance (FDR-adjusted p = 0.141). The majority of malignant tumours with viral load data (70/86, 81.4%) occurred in virally suppressed records.

#### ART regimen category.

The malignant fraction did not differ materially across regimen classes (DTG-based 49/77 [63.6%]; EFV-based 32/50 [64.0%]; other 33/50 [66.0%]; χ² p = 0.981; FDR-adjusted p = 0.981), providing no evidence that regimen class itself modifies malignancy risk in this cohort.

#### Sensitivity analysis.

Restricting the analysis to one tumour record per patient (n = 216, the earliest record by date for the three patients with two records) produced materially unchanged results for every variable in Table 3: the ranking of variables by statistical significance, the direction of every association, and the conclusions drawn were the same as in the primary tumour-record-level analysis.

### 3.5 Kaposi sarcoma versus other malignancies

Because KS is itself a malignancy, this comparison is restricted to the 138 malignant tumour records (65 KS, 73 other malignancies); it therefore uses different, smaller denominators than the malignant-versus-benign comparisons in Section 3.4 and Table 3. p-values below are FDR-adjusted within this six-comparison panel.

**Fig 4:**
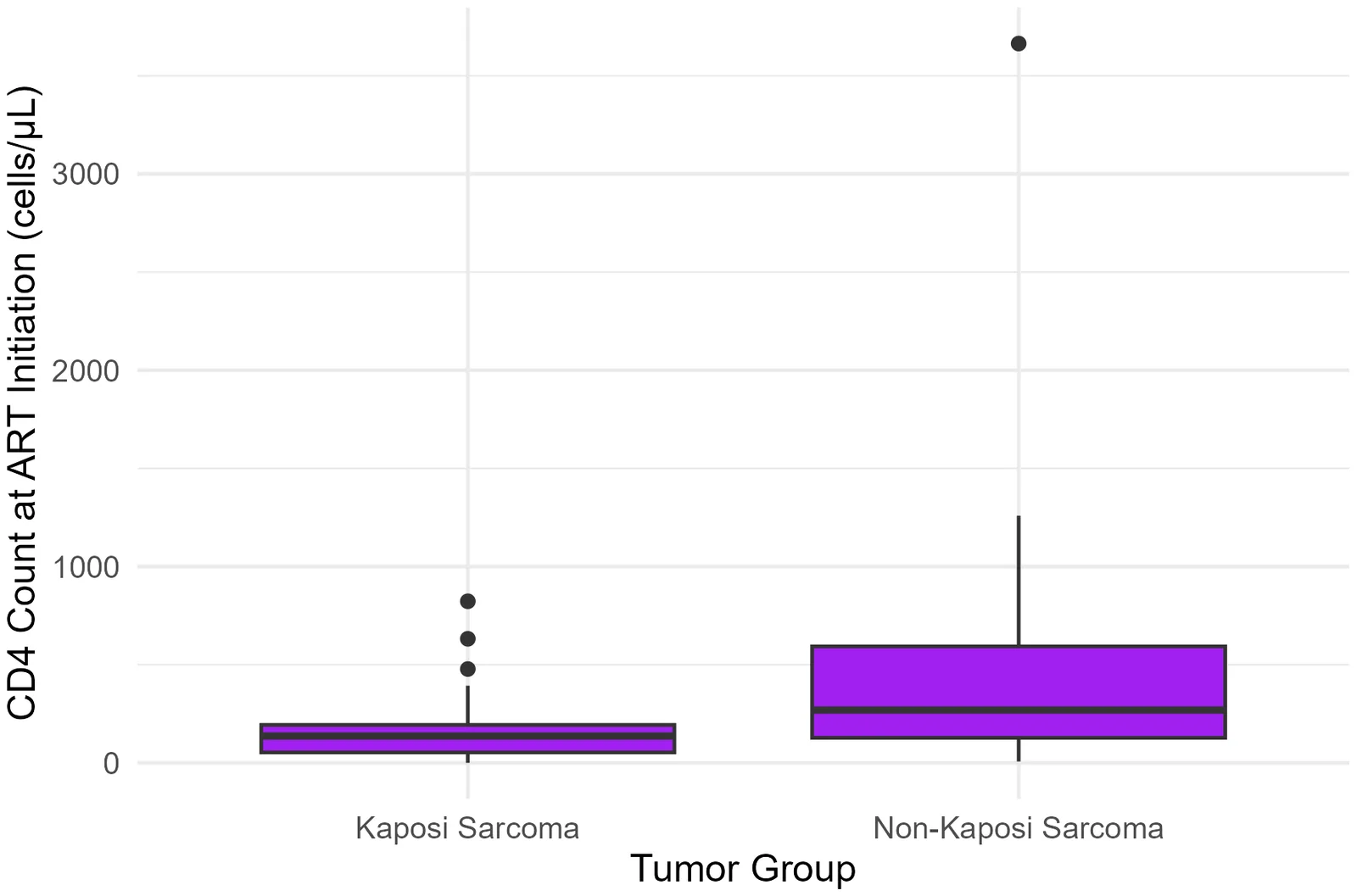
CD4 count at ART initiation: Kaposi sarcoma versus other malignancies. Box-and-whisker plots showing CD4 T-cell counts (cells/µL) at ART initiation among malignant tumour records only, stratified by Kaposi sarcoma (KS; n = 65) versus other malignancies (n = 73). This comparison is restricted to the 138 malignant tumour records so that the “other malignancies” group does not include benign tumours. Data available for 71 of 138 malignant records.

**Fig 5:**
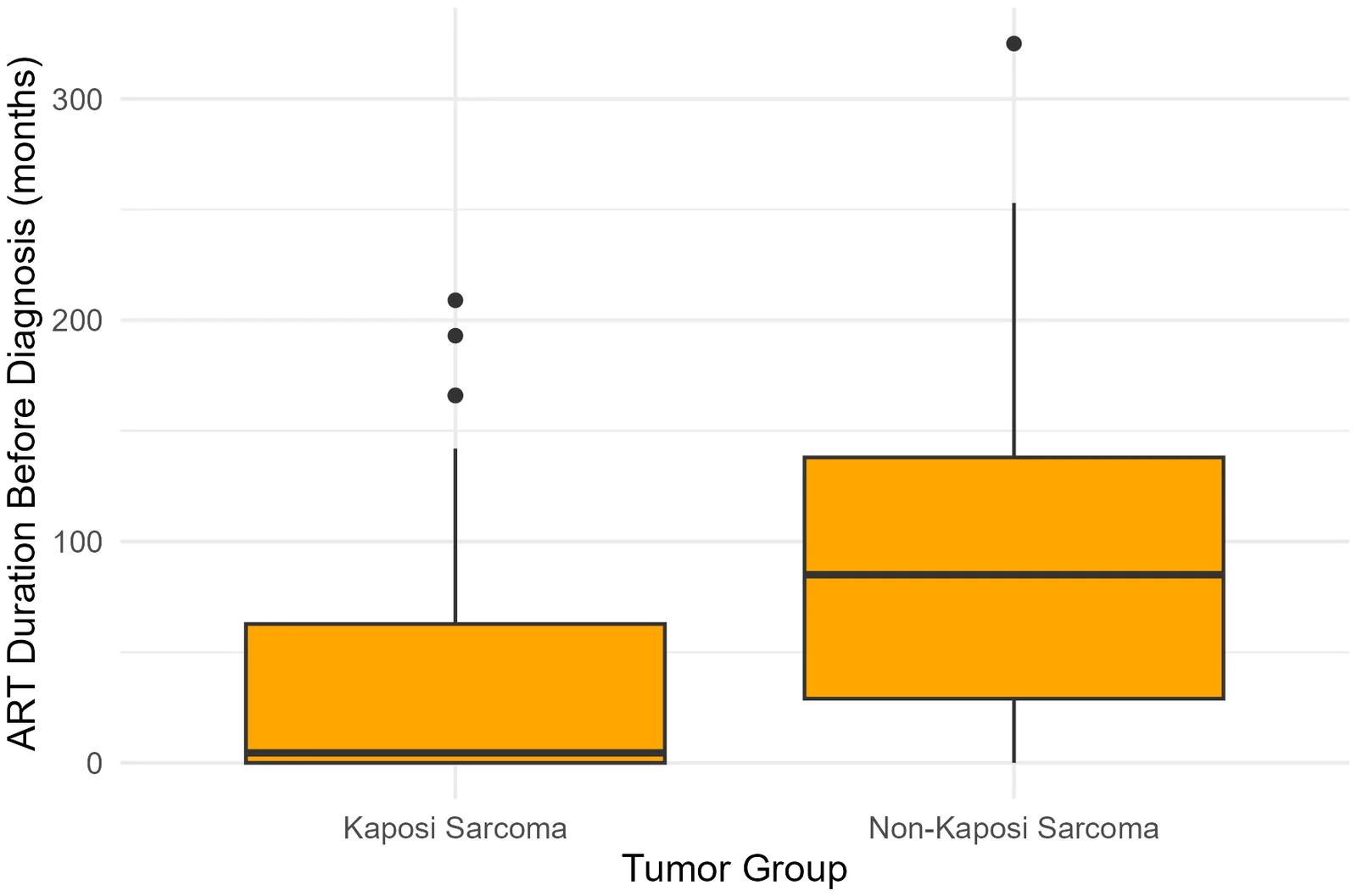
ART duration before tumour diagnosis: Kaposi sarcoma versus other malignancies. Box-and-whisker plots comparing ART duration (months) among malignant tumour records only, stratified by KS (n = 65) versus other malignancies (n = 73). KS patients had a markedly shorter median ART duration before diagnosis than patients with other malignancies (4 months, IQR 0–63, vs. 63 months, IQR 12–131; Mann–Whitney U, FDR-adjusted p < 0.001). Data available for 129 of 138 malignant records.

KS patients were younger than patients with other malignancies (median 41 vs 48 years; FDR-adjusted p = 0.041) and had markedly shorter ART duration before tumour diagnosis (4 months, IQR 0–63, vs 63 months, IQR 12–131; FDR-adjusted p < 0.001; Fig 5). Median CD4 at ART initiation was numerically lower among KS patients than those with other malignancies (136 vs 171 cells/µL) but this difference did not reach significance in this restricted, smaller-denominator comparison (n = 71; FDR-adjusted p = 0.092; Fig 4). KS patients were more likely to be male (41/65, 63.1%, vs 32/73, 43.8%; FDR-adjusted p = 0.041) and to have had advanced WHO stage at ART initiation (26/55, 47.3%, vs 13/70, 18.6%; FDR-adjusted p = 0.003) Fig 5. Viral suppression at tumour diagnosis was numerically lower among KS patients than those with other malignancies (26/36, 72.2%, vs 44/50, 88.0%) but this difference did not reach significance after FDR adjustment (p = 0.092). These findings are summarised in Table 4.

**Table 4:** Immunovirological comparison of Kaposi sarcoma versus other malignancies, restricted to malignant tumours only (n = 138).

Variable	Kaposi Sarcoma (n = 65)	Other malignancies (n = 73)	n analysed	Nominal p	FDR-adjusted p
**Age, years, median (IQR)**	41 (36–48)	48 (39–59)	138	0.021	0.041
**CD4 at ART initiation, cells/µL, median (IQR)**	136 (53–194)	171 (98–432)	71	0.079	0.092
**ART duration, months, median (IQR)**	4 (0–63)	63 (12–131)	129	<0.001	<0.001
**Male sex, n (%)**	41 (63.1)	32 (43.8)	138	0.027	0.041
**Advanced WHO stage (III/IV), n (%)^a^**	26/55 (47.3)	13/70 (18.6)	125	<0.001	0.003
**Virally suppressed at diagnosis, n (%)^a^**	26/36 (72.2)	44/50 (88.0)	86	0.092	0.092

^a^Denominator reflects malignant records with non-missing data for the variable. KS, Kaposi sarcoma; IQR, interquartile range; WHO, World Health Organization.

## 4. Discussion

This study provides an immunovirologically contextualised description of the tumour spectrum among PLHIV at a specialised HIV centre in Uganda during the transition to DTG-based ART. Four findings are central to interpreting this cohort.

### First, KS remains the single largest tumour category in this HIV clinic population, despite high measured viral suppression

KS accounted for 47.1% of all malignant tumours and 29.7% of the entire cohort. This is consistent with sub-regional cancer registry data from Uganda [14–16] and broader East African evidence [9,10], and with Kampala Cancer Registry’s age-standardised KS incidence estimate of approximately 29.6 per 100,000 [13]. We note two complementary, non-exclusive explanations for this pattern that should be weighed alongside the biological rationale. Biologically, HHV-8 seroprevalence in Ugandan adults has been reported above 40% in some population studies [29,30], HHV-8 reactivation persists in many virally suppressed PLHIV [31,32], and the immune compartments responsible for HHV-8 surveillance, notably CD8 T-cells and natural killer cells, recover incompletely following late ART initiation [33]. Separately, and important for interpreting the temporal pattern of registrations described in Section 3.1, improvements in clinical recognition and coded documentation of KS at Mildmay over the study period, particularly around the 2018 data quality programme, may also have increased ascertainment of KS relative to earlier years; we cannot fully distinguish a true rise in KS burden from improved detection and recording with the data available, and this should be tested prospectively.

### Second, viral suppression alone did not identify a low-malignancy subgroup in this cohort

The majority of malignant tumours with viral load data (81.4%) occurred in virally suppressed records, and unsuppressed viraemia was not statistically associated with malignancy after FDR adjustment (p = 0.141). This pattern is consistent with observations in high-income cohorts [4,34,35] but is less often demonstrated in routine HIV clinic data from sub-Saharan Africa. It suggests that HIV viral suppression alone does not restore immune competence against oncogenic co-infections, nor does it normalise chronic immune activation or microbial translocation pathways increasingly implicated in HIV-associated cancer biology [4,6,36]. Programmatically, viral load status alone is an insufficient basis for cancer risk stratification in this setting.

### Third, low pre-ART CD4 and advanced WHO stage were associated with malignancy at tumour diagnosis, consistent with a durable immunological footprint of late HIV diagnosis

The difference in median pre-ART CD4 (147 vs 476 cells/µL for malignant vs benign tumour records) and in the proportion with advanced WHO stage (45.3% vs 11.4%) are cross-sectional associations, not longitudinal or dose-response gradients, since each record reflects a single patient observed at one point in their care. With that caveat, these findings are consistent with the broader literature linking pre-treatment immune damage to persistent oncological vulnerability even after years of suppressive ART [37,38]. This has policy relevance for Uganda’s HIV testing and linkage programmes: earlier HIV diagnosis and ART initiation, ahead of advanced immunosuppression, plausibly reduces long-run malignancy risk, though this cohort cannot establish causality.

### Fourth, malignant tumours were disproportionately diagnosed early in the ART course, and KS in particular arose in a markedly more immunologically advanced and ART-naive-adjacent context than other malignancies

Among records diagnosed within 12 months of ART initiation, 87.1% were malignant, compared with 48.4% among those on ART for over 120 months. Two non-mutually-exclusive mechanisms are plausible: immune reconstitution inflammatory syndrome (IRIS) unmasking subclinical KS or other cancers [39], and detection of pre-existing occult malignancy during the intensified clinical engagement that accompanies ART initiation. The KS-versus-other-malignancies comparison (Table 4) sharpens this picture: KS patients had a median ART duration of only 4 months at tumour diagnosis, versus 63 months for other malignancies, and were more likely to have advanced WHO stage. Systematic tumour evaluation, including dermatological examination, lymph node assessment, and structured symptom screening, embedded within the first year of ART follow-up, is a plausible, low-cost intervention this cohort supports piloting.

Returning to the sex-stratified pattern described in Section 3.3: the higher malignant fraction among men (79.3% vs 51.2% in women) is substantially explained by the male predominance of KS (41/92 men, 44.6%, versus 24/127 women, 18.9%) combined with the high frequency of benign uterine leiomyomas among women (44/127, 34.6%, of all tumours in women), which mechanically lowers the malignant fraction in the denominator for women without implying a lower underlying malignancy risk once gynaecological screening-detected benign disease is accounted for. Gynaecological tumours were, as expected, the single largest tumour category among women; the finding we consider most informative for programme design is one that cuts across both sexes, namely that both the male-predominant KS burden and the female-predominant benign gynaecological burden coexist within the same HIV clinic population and require distinct, sex-tailored screening pathways rather than a single undifferentiated cancer-screening protocol.

The gynaecological findings also carry a distinct clinical signal: benign uterine leiomyomas constituted the largest single benign tumour category, reflecting the feminised HIV epidemic and Mildmay’s integrated women’s health services, but 10 invasive cervical cancers were identified against the backdrop of the WHO 2030 cervical cancer elimination strategy and Uganda’s HPV vaccination rollout, representing missed prevention opportunities. These findings support systematic, visit-integrated cervical screening for all women living with HIV in this setting, consistent with current guidelines [40].

### Strengths and limitations

Strengths of this study include use of a longitudinal institutional electronic record that captures HIV treatment history, virological monitoring, and ICD-10-coded tumour diagnoses within a single linked system; a near-decade-long observation window spanning a major ART policy transition; transparent, variable-specific missingness reporting; false discovery rate correction across all bivariate comparisons; a pre-specified patient-level sensitivity analysis; and a KS-versus-other-malignancies comparison correctly restricted to malignant tumour records, which is not typically reported in retrospective HIV-oncology datasets from this region.

The limitations are substantial and should temper interpretation. The retrospective, cross-sectional design, combined with non-trivial and plausibly non-random missingness for CD4 (57.5%) and viral load (29.2%), introduces potential bias; missingness for CD4 is plausibly informative, since patients on stable suppressive ART are monitored less frequently, likely creating a selection towards sicker or more recently diagnosed patients in the available-CD4 subset. The absence of a denominator of all PLHIV in care at Mildmay precludes calculation of cancer incidence rates. Reliance on ICD-10 coding without universal histological confirmation means some diagnoses may reflect clinical rather than pathological ascertainment, and the diagnostic modality could not be systematically compared across tumour types. The 2018 registration peak partly reflects a data quality programme rather than a true change in underlying cancer incidence, and we cannot fully separate improved KS ascertainment from a true change in KS burden over the study period. Three patients contributed two tumour records each; we analysed by tumour record as the pre-specified primary unit, and confirmed in a sensitivity analysis that restricting to one record per patient did not change the direction, ranking, or interpretation of any association. No multivariable regression was performed given the descriptive aim, the extent of missingness, and the resulting risk of overfitting a model with this sample size; all associations presented are unadjusted, hypothesis-generating, and should not be read as evidence of temporal or dose-response gradients.

### Implications for research and clinical practice

These findings support a programmatic research agenda at Mildmay Research Centre comprising: (i) a prospective HIV-oncology cohort with systematic tumour ascertainment, histological confirmation, and biobanking to allow HHV-8 immune profiling; (ii) a nested study of HHV-8 viral dynamics and cellular immune reconstitution trajectories among virally suppressed PLHIV with and without KS; (iii) integration of HPV-based cervical screening and screen-and-treat into routine HIV care for all women; and (iv) implementation research on the yield, cost, and acceptability of structured cancer symptom screening embedded in early ART follow-up visits. Each priority is operationally feasible at our centre.

## 5. Conclusions

Among PLHIV attending a specialised HIV centre in Uganda, tumours identified through routine clinical care are predominantly malignant and dominated by Kaposi sarcoma, despite high measured viral suppression. Malignancy is associated with low CD4 at ART initiation, advanced WHO stage, and short ART duration in unadjusted, cross-sectional comparisons that are hypothesis-generating rather than causal. KS arises in a distinctly different immunovirological context than other malignancies in this cohort, with markedly shorter ART duration and higher WHO stage at ART initiation. These data support a tractable clinical research and surveillance agenda: cancer-symptom vigilance for all PLHIV regardless of current viral load status; structured tumour evaluation during the early ART period; integration of cervical cancer screening into routine HIV care; and a prospective HIV-oncology cohort to disentangle the residual mechanisms, immune senescence, incomplete reconstitution, oncoviral co-infection, and chronic inflammation, that plausibly sustain HIV-associated cancer risk in virally suppressed patients.

## References

[1] Engels EA, Biggar RJ, Hall HI, Cross H, Crutchfield A, Finch JL, et al. Cancer risk in people infected with human immunodeficiency virus in the United States. Int J Cancer. 2008;123(1):187–94. doi: 10.1002/ijc.23487 18435450

[2] Grulich AE, van Leeuwen MT, Falster MO, Vajdic CM. Incidence of cancers in people with HIV/AIDS compared with immunosuppressed transplant recipients: a meta-analysis. Lancet. 2007;370(9581):59–67. doi: 10.1016/S0140-6736(07)61050-2 17617273

[3] Shiels MS, Engels EA. Evolving epidemiology of HIV-associated malignancies. Curr Opin HIV AIDS. 2017;12(1):6–11. doi: 10.1097/COH.0000000000000327 27749369 PMC5240042

[4] Hernández-Ramírez RU, Shiels MS, Dubrow R, Engels EA. Cancer risk in HIV-infected people in the USA from 1996 to 2012: a population-based, registry-linkage study. Lancet HIV. 2017;4(11):e495–504. doi: 10.1016/S2352-3018(17)30125-X 28803888 PMC5669995

[5] Robbins HA, Pfeiffer RM, Shiels MS, Li J, Hall HI, Engels EA. Excess cancers among HIV-infected people in the United States. J Natl Cancer Inst. 2015;107(4):dju503. doi: 10.1093/jnci/dju503 25663691 PMC4334816

[6] Borges AH, Dubrow R, Silverberg MJ. Factors contributing to risk for cancer among HIV-infected individuals, and evidence that earlier combination antiretroviral therapy will alter this risk. Curr Opin HIV AIDS. 2014;9(1):34–40. doi: 10.1097/COH.0000000000000025 24225382 PMC3992916

[7] Mwakigonja AR, Pyakurel P, Kokhaei P, Pak F, Lema LK, Kaaya EE, et al. Human herpesvirus-8 (HHV-8) sero-detection and HIV association in Kaposi’s sarcoma (KS), non-KS tumors and non-neoplastic conditions. Infect Agent Cancer. 2008;3:10. doi: 10.1186/1750-9378-3-10 18590556 PMC2499990

[8] Casper C. The increasing burden of HIV-associated malignancies in resource-limited regions. Annu Rev Med. 2011;62:157–70. doi: 10.1146/annurev-med-050409-103711 20868276

[9] Mosam A, Shaik F, Uldrick TS, Esterhuizen T, Friedland GH, Scadden DT, et al. A randomised controlled trial of HAART versus HAART and chemotherapy in therapy-naïve HIV-associated Kaposi sarcoma in South Africa. J Acquir Immune Defic Syndr. 2012;60(2):150–7. 10.1097/QAI.0b013e318251aedd PMC3360837 22395672

[10] Bohlius J, Valeri F, Maskew M, Prozesky H, Garone D, Sengayi M, et al. Kaposi’s Sarcoma in HIV-infected patients in South Africa: multicohort study in the antiretroviral therapy era. Int J Cancer. 2014;135(11):2644–52. doi: 10.1002/ijc.28894 24729433 PMC4415728

[11] Parkin DM, Sitas F, Chirenje M, Stein L, Abratt R, Wabinga H. Part I: cancer in indigenous africans--burden, distribution, and trends. Lancet Oncol. 2008;9(7):683–92. doi: 10.1016/S1470-2045(08)70175-X 18598933

[12] Sung H, Ferlay J, Siegel RL, Laversanne M, Soerjomataram I, Jemal A, et al. Global cancer statistics 2020: GLOBOCAN estimates of incidence and mortality worldwide for 36 cancers in 185 countries. CA Cancer J Clin. 2021;71(3):209–49. 10.3322/caac.21660 33538338

[13] Asasira J, Lee S, Tran TXM, Mpamani C, Wabinga H, Jung S-Y, et al. Infection-related and lifestyle-related cancer burden in Kampala, Uganda: projection of the future cancer incidence up to 2030. BMJ Open. 2022;12(3):e056722. doi: 10.1136/bmjopen-2021-056722 35296484 PMC8928275

[14] Wabinga HR, Nambooze S, Amulen PM, Okello C, Mbus L, Parkin DM. Trends in the incidence of cancer in Kampala, Uganda, 1991–2010. Int J Cancer. 2014;135(2):432–9. 10.1002/ijc.28661 24615279

[15] Okuku F, Krantz EM, Kafeero J, Kamya MR, Orem J, Casper C, et al. Evaluation of a Predictive Staging Model for HIV-Associated Kaposi Sarcoma in Uganda. J Acquir Immune Defic Syndr. 2017;74(5):548–54. doi: 10.1097/QAI.0000000000001286 28107226 PMC5340582

[16] Stoeter O, Seraphin TP, Chitsike I, Chokunonga E, Kambugu JB, Wabinga H, et al. Trends in childhood cancer incidence in sub-Saharan Africa: results from 25 years of cancer registration in Harare (Zimbabwe) and Kyadondo (Uganda). Int J Cancer. 2021;149(5):1002–12. doi: 10.1002/ijc.33619 33945631

[17] World Health Organization. Updated recommendations on first-line and second-line antiretroviral regimens. Geneva: WHO; 2019.

[18] Uganda Ministry of Health. Consolidated guidelines for the prevention and treatment of HIV and AIDS in Uganda. Kampala: MoH; 2020.

[19] Zakumumpa H, Kitutu FE, Ndagije HB, Diana N-K, Ssanyu JN, Kiguba R. Provider perspectives on the acceptability and tolerability of dolutegravir-based anti-retroviral therapy after national roll-out in Uganda: a qualitative study. BMC Infect Dis. 2021;21(1):1222. doi: 10.1186/s12879-021-06933-8 34876050 PMC8650263

[20] Mwaka AD, Garimoi CO, Were EM, Roland M, Wabinga H, Lyratzopoulos G. Social, demographic and healthcare factors associated with stage at diagnosis of cervical cancer: cross-sectional study in a tertiary hospital in Northern Uganda. BMJ Open. 2016;6(1):e007690. doi: 10.1136/bmjopen-2015-007690 26801459 PMC4735146

[21] Stewart EA, Cookson CL, Gandolfo RA, Schulze-Rath R. Epidemiology of uterine fibroids: a systematic review. BJOG. 2017;124(10):1501–12. doi: 10.1111/1471-0528.14640 28296146

[22] Lim KB. Epidemiology of clinical benign prostatic hyperplasia. Asian J Urol. 2017;4(3):148–51. doi: 10.1016/j.ajur.2017.06.004 29264223 PMC5717991

[23] Chireshe R, Manyangadze T, Naidoo K. Integrated chronic care models for people with comorbid of HIV and non-communicable diseases in Sub-Saharan Africa: a scoping review. PLoS One. 2024;19(3):e0299904. doi: 10.1371/journal.pone.0299904 38489252 PMC10942093

[24] Patel P, Rose CE, Collins PY, Nuche-Berenguer B, Sahasrabuddhe VV, Peprah E, et al. Noncommunicable diseases among HIV-infected persons in low-income and middle-income countries: a systematic review and meta-analysis. AIDS. 2018;32 Suppl 1(Suppl 1):S5–20. doi: 10.1097/QAD.0000000000001888 29952786 PMC6380891

[25] Uganda Ministry of Health. National HIV testing services policy and implementation guidelines. 4th ed. Kampala: MoH; 2022.

[26] World Health Organization. International statistical classification of diseases and related health problems, 10th revision (ICD-10). Geneva: WHO; 2019.

[27] World Medical Association. World Medical Association Declaration of Helsinki: ethical principles for medical research involving human subjects. JAMA. 2013;310(20):2191–4. doi: 10.1001/jama.2013.281053 24141714

[28] von Elm E, Altman DG, Egger M, Pocock SJ, Gøtzsche PC, Vandenbroucke JP, et al. The Strengthening the Reporting of Observational Studies in Epidemiology (STROBE) statement: guidelines for reporting observational studies. Lancet. 2007;370(9596):1453–7. doi: 10.1016/S0140-6736(07)61602-X 18064739

[29] Newton R, Labo N, Wakeham K, Baisley K, Mayanja Y, Genberg B, et al. Kaposi’s sarcoma-associated herpesvirus in a rural Ugandan cohort, 1992–2008. J Infect Dis. 2018;217(2):263–9. 10.1093/infdis/jix569 PMC5853224 29099933

[30] Wakeham K, Webb EL, Sebina I, Nalwoga A, Muhangi L, Miley W, et al. Risk factors for seropositivity to Kaposi sarcoma-associated herpesvirus among children in Uganda. J Acquir Immune Defic Syndr. 2013;63(2):228–33. doi: 10.1097/QAI.0b013e31828a7056 23403859 PMC3707567

[31] Lidenge SJ, Kossenkov AV, Tso FY, Wickramasinghe J, Privatt SR, Ngalamika O, et al. Comparative transcriptome analysis of endemic and epidemic Kaposi’s sarcoma (KS) lesions and the secondary role of HIV-1 in KS pathogenesis. PLoS Pathog. 2020;16(7):e1008681. doi: 10.1371/journal.ppat.1008681 32706839 PMC7406108

[32] Polizzotto MN, Uldrick TS, Wyvill KM, Aleman K, Marshall VA, Wang V, et al. Clinical features and outcomes of patients with symptomatic Kaposi sarcoma herpesvirus inflammatory cytokine syndrome. Clin Infect Dis. 2016;62(6):730–8. 10.1093/cid/civ996 PMC4772848 26658701

[33] Cao W, Mehraj V, Kaufmann DE, Li T, Routy J-P. Elevation and persistence of CD8 T-cells in HIV infection: the Achilles heel in the ART era. J Int AIDS Soc. 2016;19(1):20697. doi: 10.7448/IAS.19.1.20697 26945343 PMC4779330

[34] Park LS, Tate JP, Sigel K, Brown ST, Crothers K, Gibert C, et al. Association of Viral suppression with lower AIDS-defining and non-AIDS-defining cancer incidence in HIV-infected veterans: a prospective cohort study. Ann Intern Med. 2018;169(2):87–96. doi: 10.7326/M16-2094 29893768 PMC6825799

[35] Hleyhel M. Risk of non-AIDS-defining cancers among HIV-1-infected individuals in France between 1997 and 2009. AIDS. 2014;28(14):2109–18. doi: 10.1097/qad.0000000000000382 25265077

[36] Deeks SG, Tracy R, Douek DC. Systemic effects of inflammation on health during chronic HIV infection. Immunity. 2013;39(4):633–45. doi: 10.1016/j.immuni.2013.10.001 24138880 PMC4012895

[37] Silverberg MJ, Chao C, Leyden WA, Xu L, Horberg MA, Klein D, et al. HIV infection, immunodeficiency, viral replication, and the risk of cancer. Cancer Epidemiol Biomarkers Prev. 2011;20(12):2551–9. doi: 10.1158/1055-9965.EPI-11-0777 22109347 PMC3237725

[38] Yanik EL, Napravnik S, Cole SR, Achenbach CJ, Gopal S, Olshan A, et al. Incidence and timing of cancer in HIV-infected individuals following initiation of combination antiretroviral therapy. Clin Infect Dis. 2013;57(5):756–64. doi: 10.1093/cid/cit369 23735330 PMC3739467

[39] Achenbach CJ, Harrington RD, Dhanireddy S, Crane HM, Casper C, Kitahata MM. Paradoxical immune reconstitution inflammatory syndrome in HIV-infected patients treated with combination antiretroviral therapy. Clin Infect Dis. 2012;54(3):424–33. 10.1093/cid/cir802 PMC3258272 22095568

[40] World Health Organization. Global strategy to accelerate the elimination of cervical cancer as a public health problem. Geneva: WHO; 2020.

