# Supplementary material for: Spectrum and immunovirological determinants of tumours among people living with HIV in Uganda: A retrospective cross sectional study from a specialised HIV centre, 2017–2026
Source: PLoS One. 2026 Sep 22;21(9):e0358066. doi: 10.1371/journal.pone.0358066 (PMC13596795; doi:10.1371/journal.pone.0358066)
Supplement: Peer review history

# Peer review: Decision Letter 0

**DOI:** 10.1371/journal.pone.0358066.r001

**Authors:** Felix Bongomin

29 Jul 2026

Dear Dr. Mwaka,

Thank you for submitting your manuscript to PLOS ONE. After careful consideration, we feel that it has merit but does not fully meet PLOS ONE’s publication criteria as it currently stands. Therefore, we invite you to submit a revised version of the manuscript that addresses the points raised during the review process.

Please submit your revised manuscript by Sep 27 2026 11:59PM. If you will need more time than this to complete your revisions, please reply to this message or contact the journal office at plosone@plos.org. When you're ready to submit your revision, log on to https://www.editorialmanager.com/pone/ and select the 'Submissions Needing Revision' folder to locate your manuscript file.

- A letter that responds to each point raised by the academic editor and reviewer(s). You should upload this letter as a separate file labeled 'Response to Reviewers'.
- A marked-up copy of your manuscript that highlights changes made to the original version. You should upload this as a separate file labeled 'Revised Manuscript with Track Changes'.
- An unmarked version of your revised paper without tracked changes. You should upload this as a separate file labeled 'Manuscript'.

If you would like to make changes to your financial disclosure, please include your updated statement in your cover letter. Guidelines for resubmitting your figure files are available below the reviewer comments at the end of this letter.

If applicable, we recommend that you deposit your laboratory protocols in protocols.io to enhance the reproducibility of your results. Protocols.io assigns your protocol its own identifier (DOI) so that it can be cited independently in the future. For instructions see: https://journals.plos.org/plosone/s/submission-guidelines#loc-laboratory-protocols. Additionally, PLOS ONE offers an option for publishing peer-reviewed Lab Protocol articles, which describe protocols hosted on protocols.io. Read more information on sharing protocols at https://plos.org/protocols?utm_medium=editorial-email&utm_source=authorletters&utm_campaign=protocols.

As the corresponding author, your ORCID iD is verified in the submission system and will appear in the published article. PLOS supports the use of ORCID, and we encourage all coauthors to register for an ORCID iD and use it as well. Please encourage your coauthors to verify their ORCID iD within the submission system before final acceptance, as unverified ORCID iDs will not appear in the published article. *Only*  the individual author can complete the verification step; PLOS staff *cannot*  verify ORCID iDs on behalf of authors.

We look forward to receiving your revised manuscript.

Kind regards,

Felix Bongomin, MB ChB, Ph.D., FECMM

Academic Editor

PLOS One

Journal Requirements:

When submitting your revision, we need you to address these additional requirements.

1. Please ensure that your manuscript meets PLOS ONE's style requirements, including those for file naming. The PLOS ONE style templates can be found at https://journals.plos.org/plosone/s/file?id=wjVg/PLOSOne_formatting_sample_main_body.pdf and https://journals.plos.org/plosone/s/file?id=ba62/PLOSOne_formatting_sample_title_authors_affiliations.pdf

2. In the online submission form you indicate that your data is not available for proprietary reasons and have provided a contact point for accessing this data. Please note that your current contact point is a co-author on this manuscript. According to our Data Policy, the contact point must not be an author on the manuscript and must be an institutional contact, ideally not an individual. Please revise your data statement to a non-author institutional point of contact, such as a data access or ethics committee, and send this to us via return email. Please also include contact information for the third-party organization, and please include the full citation of where the data can be found.

3. Your ethics statement should only appear in the Methods section of your manuscript. If your ethics statement is written in any section besides the Methods, please delete it from any other section.

If the reviewer comments include a recommendation to cite specific previously published works, please review and evaluate these publications to determine whether they are relevant and should be cited. There is no requirement to cite these works unless the editor has indicated otherwise.

[Note: HTML markup is below. Please do not edit.]

Reviewers' comments:

Reviewer's Responses to Questions

**Comments to the Author**

1. Is the manuscript technically sound, and do the data support the conclusions?

Reviewer #1: Partly

**********

2. Has the statistical analysis been performed appropriately and rigorously?

Reviewer #1: No

**********

3. Have the authors made all data underlying the findings in their manuscript fully available?

The PLOS Data policy

Reviewer #1: No

**********

4. Is the manuscript presented in an intelligible fashion and written in standard English?

Reviewer #1: Yes

**********

Reviewer #1: Abstract

Background.

The first sentence is long and confusing. The authors could break it down and make it clearer.

Methods

What does the author mean by “unique people living with HIV”?

Results

Female percentage is missing the number of females.

Main article

“With the advent of sustained viral suppression, the relative burden of AIDS-defining cancers declined in high-income settings, while non-AIDS-defining cancers (NADCs) including hepatocellular, lung, anal, and colorectal carcinomas rose” For that statement, is it the incidence or prevalence? Secondly could you breakdown that sentence into two parts.

“KS remains the most frequently reported cancer among PLHIV across East Africa, reflecting an exceptionally high regional seroprevalence of HHV-8 (often exceeding 40% in adult populations) and persistent immune reconstitution gaps that follow late ART initiation.” What kind of quantative information are you trying to report here? KS remains most frequently report in terms of?

Uganda carries one of the highest age-standardised KS incidence rates globally. This needs to be referenced and the figure stated.

Please indicate when DTG was introduced in Uganda and the currently coverage as you argument is based on that.

Methods

The Mildmay Research Centre which is the Research arm of Mildmay Uganda is located within the same compass with Mildmay Hospital. Please correct the grammar in this sentence.

The authors could clarify if the study was retrospective observational cohort study or a cross-sectional study nested in a cohort study. As per their description, the study appears to be cross-sectional in nature.

The authors noted that their primary outcome was presence of tumor irrespective of how the diagnosis was made. They could clarify how Mildmay makes the diagnosis of the tumor and what was consistent in this group.

Is there a reason as to why age was categorised? The same goes for ART duration. Was the data nonparametric?

They should define WHO.

The authors can remove the word “prevailing” before Ugandan Ministry of health. It is misused in that context. Same goes for ART backbone.

It is important that the authors highlight the missing values or entries, how many they were and if they used complete case analysis, how did this impact their analysis.

Define what OR is.

Please describe how you addressed the issues of multiple comparison and why tumor record was the primary unit of analysis given that some participants had two. How was it handled?

Results

What is unique about the PHLIV group?

No need for the range.

Wouldn’t it be expected that Gynaecological tumors would be the most reported among females? The important finding in the sex stratified pattern would be something that applies to both men and women. “Driven primarily by the male predominance of KS and the high frequency of benign uterine fibroids in women.” This should be in your discussion not results.

This is unclear” The gradient was striking among the 25 patients in WHO stage IV at ART initiation, 24 (96.0%) were subsequently diagnosed with a malignant tumour, of whom 20 (83.3%) had KS. This represents a near-complete enrichment of malignancy among the most immunologically compromised patients at ART entry.” You plotted a histogram without a gradient. Where does the gradient you are hitting on in this result come from?

The author needs to rephrase this “The categorical analysis reinforced this gradient: 44/71 (62.0%) patients with pre-ART CD4…..” The statement implies something else not supported by the results. Secondly, the figure quoted had no gradiate and based on how the data was collected, there is no longitudinal linkage between CD4 count and presence of tumor.

Still, inappropriate phrasing that does not support the data “Malignancy risk was heavily concentrated in the earliest ART period: 54/62 (87.1%) of patients diagnosed within ≤12 months of ART initiation had a malignant tumour, declining to 31/64 (48.4%) among those on ART for >120 months (Figure 3). This temporal gradient supports an interpretation of both unmasking IRIS-associated KS and detection of previously occult malignancies during early clinical engagement.”

Still “Although unsuppressed viraemia was numerically associated with a higher malignant proportion (16/22, 72.7% vs 70/134, 52.2%; OR 2.44, 95% CI 0.90–6.61), this difference did not reach statistical significance (Fisher exact p=0.105). Critically, the absolute majority of malignant tumours (70/86, 81.4%) occurred in virally suppressed patients, a pattern that underscores the insufficiency of viral suppression alone as a cancer risk stratification tool.” The study did not study viral load as a continuous variable. It becomes difficult to support the wording of the first statement as the nature of the data collected doesn’t support (frequency). Secondly, I would refrain from using numerically associated. You could highlight that more people with viral suppression at time t had this when compared to this, but that was not statistically significant.

Using association wording for crude and unadjusted estimate might confuse readers. Since no regression analysis was conducted in this study, I would refrain from using association.

See my comment above for this “KS patients were also significantly more likely to be male (41/65, 63.1% vs 32/73, 43.8%; p=0.024) and to have had advanced WHO stage at ART initiation (65.8% vs 29.2% with available data; p=0.001). Interestingly, viral suppression rates at the time of tumour diagnosis did not differ between KS and other malignancies (81.3% vs 81.6%; p=0.97), reinforcing the point that KS in Uganda frequently arises in virally suppressed but immunologically vulnerable individuals. These findings are summarised in Table 4 and illustrated in Figures 4 and 5.”

From your background and research question, “emporal pattern of tumour registrations Annual tumour registrations peaked in 2018 (n=73), reflecting a data quality improvement programme, then settled to 10–19 records per year between 2020 and 2024, with a modest increase to 18 in 2025. The pre-2020 peak should not be interpreted as a true epidemiological surge in cancer incidence.” This is not required.

Discussion:

In this discussion, it is not clear what the key findings are. The author hints on the fact that most of the people in this study had Kaposi Sacroma but fails to highlight the fact that screening and diagnosis for KS might have improved during this period. He alludes to the biological mechanism of the disease which is well known and established but fails to discuss the results in the context of Mildmay or Uganda. I think the author needs to re-evaluate the results and come up with a discussion tailored to his results that is clear and less redundant.

**********

what does this mean?). If published, this will include your full peer review and any attached files.

If you choose “no”, your identity will remain anonymous but your review may still be made public.

**Do you want your identity to be public for this peer review?**  For information about this choice, including consent withdrawal, please see our Privacy Policy

Reviewer #1: No

**********

[NOTE: If reviewer comments were submitted as an attachment file, they will be attached to this email and accessible via the submission site. Please log into your account, locate the manuscript record, and check for the action link "View Attachments". If this link does not appear, there are no attachment files.]

To ensure your figures meet our technical requirements, please review our figure guidelines: https://journals.plos.org/plosone/s/figures

You may also use PLOS’s free figure tool, NAAS, to help you prepare publication quality figures: https://journals.plos.org/plosone/s/figures#loc-tools-for-figure-preparation.

NAAS will assess whether your figures meet our technical requirements by comparing each figure against our figure specifications.

# Author comment: Author response to Decision Letter 1

**DOI:** 10.1371/journal.pone.0358066.r002

14 Aug 2026

Manuscript: PONE-D-26-21762

Title: Spectrum and Immunovirological Determinants of Tumours Among People Living with HIV in Uganda: A Retrospective Cross Sectional Study from a Specialised HIV Centre, 2017–2026

Corresponding author: Dr. Joseph Mwaka, Mildmay Research Centre, Kampala, Uganda

We thank the Academic Editor and Reviewer 1 for their careful and constructive appraisal of our manuscript. The comments have substantially improved the work. In preparing this revision we re-ran the entire analysis from the source dataset, which also allowed us to correct several data-handling issues; as a result, some numbers differ slightly from the original submission (the analytic dataset is now 219 tumour records from 216 patients, after excluding one record with an uninterpretable WHO clinical stage value; see our response to the Methods comments below). All figures, tables, and in-text values have been regenerated consistently.

Below, each editor and reviewer comment is reproduced in bold, followed by our response and, where applicable, the location of the change in the revised manuscript. Line and section numbers refer to the clean (unmarked) revised manuscript.

A. Journal Requirements (Academic Editor)

1. Please ensure that your manuscript meets PLOS ONE’s style requirements, including those for file naming.

Response: We have reformatted the manuscript to follow the PLOS ONE style templates for the main body and for the title/authors/affiliations page. Files are named per PLOS ONE convention (Manuscript, Response to Reviewers, Revised Manuscript with Track Changes). Figures are provided as separate files, named Fig 1 through Fig 5, and are cited in the text as “Fig 1” etc.; figure legends are listed together after the reference list, as required.

2. The data availability contact point must not be an author and must be an institutional contact.

Response: We have revised the Data Availability statement so that the contact point is the Mildmay Uganda Research Ethics Committee (MUREC), an institutional body that is independent of all authors, rather than a co-author. The revised statement (Declarations, “Availability of data and materials”) reads: the de-identified data underlying the findings are available upon reasonable request to MUREC, citing protocol MUREC-2025-808. We have provided the specific institutional MUREC contact address in the online submission form and in this statement. We have incorporated the revised data statement in the revised manuscript.

3. Your ethics statement should only appear in the Methods section.

Response: We have removed the ethics statement from the Declarations section at the end of the manuscript. The ethics statement now appears only once, in the Methods (Section 2.6, “Ethical considerations”). The Declarations section retains only the data-availability, competing-interests, funding, contributions, and acknowledgements items.

B. Reviewer 1: Abstract

Background. The first sentence is long and confusing. The authors could break it down and make it clearer.

Response: We have split the opening of the Abstract background into three shorter sentences (Abstract, Background). The first now states the burden; the second the tumour-spectrum gap; the third the immunovirological gap.

Methods. What does the author mean by “unique people living with HIV”?

Response: We agree this phrasing was ambiguous. We have removed the word “unique” throughout. The Abstract and Methods now state plainly that the 219 tumour records were contributed by 216 individual patients, three of whom each had two separate tumour diagnoses on different dates (Abstract, Methods; Methods Section 2.2).

Results. Female percentage is missing the number of females.

Response: Corrected. The Abstract and all relevant places now give the numerator with every percentage, for example “127/219 (58.0%) female” (Abstract, Results; Table 1; Table 3). Every statistic in Tables 1, 3, and 4 now reports its own explicit denominator.

C. Reviewer 1: Introduction / Main article

“With the advent of sustained viral suppression, the relative burden of AIDS-defining cancers declined … while non-AIDS-defining cancers (NADCs) … rose.” Is it incidence or prevalence? Break the sentence into two.

Response: We have clarified that we are referring to incidence, and split the sentence in two (Introduction, paragraph 1). The revised text states that the relative incidence of AIDS-defining cancers has declined in high-income settings, and then, in a separate sentence, that the incidence of NADCs has risen, listing the contributing factors.

“KS remains the most frequently reported cancer among PLHIV across East Africa …” What quantitative information is being reported? KS remains most frequently reported in terms of?

Response: We have made the quantitative referent explicit. The revised sentence states that KS is the single most frequently reported cancer type among registered malignancies in PLHIV across East Africa, and separately reports the regional HHV-8 seroprevalence figure (over 40% in some adult population studies) as the cited mechanistic context, rather than implying that the seroprevalence figure quantifies KS frequency (Introduction, paragraph 2).

“Uganda carries one of the highest age-standardised KS incidence rates globally.” This needs to be referenced and the figure stated.

Response: We have added the figure and a supporting reference. The revised text states that Uganda’s Kampala Cancer Registry has recorded an age-standardised KS incidence of approximately 29.6 per 100,000, among the highest reported for any population-based registry, and cites Asasira et al. (BMJ Open 2022), which reports this figure from Kampala Cancer Registry data (Introduction, paragraph 2; reference 13). We also note the declining trajectory of this rate, for balance.

Please indicate when DTG was introduced in Uganda and the current coverage, as your argument is based on that.

Response: We have added this. The revised Introduction states that Uganda adopted DTG-based ART as its preferred first-line regimen in 2018, with phased national roll-out beginning that year following WHO 2018–2019 guidance and cites a Uganda-specific source documenting the national roll-out (Zakumumpa et al., BMC Infect Dis 2021; reference 18) alongside the WHO and Ministry of Health guideline references (Introduction, paragraph 3; references 16, 17, 18).

D. Reviewer 1: Methods

“The Mildmay Research Centre which is the Research arm of Mildmay Uganda is located within the same compass with Mildmay Hospital.” Please correct the grammar.

Response: Corrected. The sentence now reads: “Mildmay Research Centre, the research arm of Mildmay Uganda, is co-located with Mildmay Hospital.” (Methods Section 2.1).

Clarify if the study was a retrospective observational cohort study or a cross-sectional study nested in a cohort. As described, it appears cross-sectional.

Response: We agree and have reframed the study accordingly. Section 2.1 now states that the analysis draws on tumour records identified retrospectively within the longitudinal clinical cohort of PLHIV in continuous care at Mildmay, but that, because a full at-risk denominator and person-time were not available, incidence could not be calculated and the comparative analyses are cross-sectional in design (they evaluate characteristics recorded at ART initiation and at tumour diagnosis rather than following individuals prospectively). We have made this framing consistent throughout, including in the title’s “Retrospective Cohort Study” being supported in the text by an explicit statement of the cross-sectional nature of the comparisons. We also draw on this reframing to temper all “gradient” and temporal language (see our responses under Results below).

Clarify how Mildmay makes the diagnosis of the tumour and what was consistent in this group.

Response: We have added detail on diagnosis (Methods Section 2.2). Diagnoses were made through routine clinical care using the modality appropriate to the presenting tumour (for example, histopathology for accessible lesions such as cutaneous KS or cervical biopsies, and clinical or radiological criteria for others such as hepatocellular carcinoma or CNS tumours). We also state honestly that the specific diagnostic modality was not uniformly recorded across tumour types and therefore could not be systematically compared, and we carry this forward as a limitation.

Is there a reason age was categorised? The same for ART duration. Was the data nonparametric?

Response: We have clarified this (Methods Sections 2.3 and 2.5). Age and ART duration were categorised into clinically standard bands for descriptive table presentation only; all inferential comparisons of these variables used the untransformed continuous measure with non-parametric tests. We added Shapiro–Wilk testing, which showed CD4 and ART duration departed significantly from normality (both p<0.001), justifying the non-parametric approach; age was closer to normal (p=0.39) but was analysed with the same non-parametric test for methodological consistency, which we now state explicitly.

They should define WHO.

Response: Defined. WHO is expanded to World Health Organization at first use, and the WHO clinical staging system is briefly defined where WHO clinical stage is introduced (Methods Section 2.3).

Remove the word “prevailing” before Uganda Ministry of Health. Same for ART backbone.

Response: Both instances of “prevailing” have been removed. The viral load threshold sentence now reads “per the Uganda Ministry of Health threshold in use during the study period” (Methods Section 2.3).

Highlight the missing values or entries, how many, and if complete case analysis was used, how it impacted the analysis.

Response: We have substantially expanded the treatment of missingness (Methods Sections 2.4 and 2.5; new detail in Results Section 3.1). We now report the exact count and percentage missing for every analysis variable (CD4 126/219, 57.5%; viral load 64/219, 29.2%; ART regimen 42/219, 19.2%; WHO stage 15/219, 6.8%; ART duration 9/219, 4.1%; age and sex 0%). We state that all analyses used complete cases for each specific variable, that every statistic in the manuscript reports its own denominator, and that CD4 missingness is plausibly not missing completely at random (patients stable on suppressive ART, or those transferring in after ART initiation elsewhere, are less likely to have a contemporaneous CD4 on file). We discuss the resulting potential for selection bias in the Limitations.

Define what OR is.

Response: Odds ratio is now defined at first use in the Methods, including a plain-language description (“the ratio of the odds of malignancy in one group relative to a reference group”) and a note that all ORs reported are crude/unadjusted (Methods Section 2.5).

Describe how you addressed multiple comparisons, and why the tumour record was the primary unit of analysis given that some participants had two. How was it handled?

Response: Both points are now addressed explicitly (Methods Section 2.5).

For multiple comparisons: we applied the Benjamini–Hochberg false discovery rate (FDR) procedure across each panel of bivariate tests (the malignant-versus-benign panel and the KS-versus-other-malignancies panel were corrected separately). Both nominal and FDR-adjusted p-values are now reported in Tables 3 and 4 and in the Results text. The substantive conclusions were unchanged after adjustment.

For the unit of analysis: we state that the tumour record was chosen as the primary unit because the study’s focus is the tumour spectrum rather than patient-level incidence, and because only 3 of 216 patients (1.4%) contributed more than one record. To confirm this choice did not drive the results, we added a pre-specified sensitivity analysis restricted to one record per patient (the earliest by date; n=216). Every association in Table 3 was materially unchanged in direction, ranking, and significance; this is reported in Results Section 3.4 and the full comparison is provided as S2 Table.

E. Reviewer 1: Results

What is unique about the PLHIV group?

Response: As above, we have removed the word “unique” throughout and now simply state the number of patients and records (Results Section 3.1).

No need for the range.

Response: We have removed the age range (7–87) that previously followed the median; the cohort age is now reported as median 47 (IQR 39–54) only (Results Section 3.1; Abstract).

Wouldn’t it be expected that gynaecological tumours would be the most reported among females? The important finding in the sex-stratified pattern would apply to both men and women. “Driven primarily by the male predominance of KS and the high frequency of benign uterine fibroids in women” should be in the Discussion, not Results.

Response: We agree on both points. In Results (Section 3.3), we now report the sex-stratified pattern descriptively and neutrally, noting that gynaecological tumours were, as expected, the largest category among women. The interpretive sentence about male-predominant KS and female-predominant benign fibroids has been moved to the Discussion (Section 4), where we now frame the cross-cutting point the reviewer identifies: that the male KS burden and the female benign gynaecological burden coexist in the same clinic population and call for distinct, sex-tailored screening pathways rather than a single undifferentiated protocol.

“The gradient was striking among the 25 patients in WHO stage IV … near-complete enrichment …” You plotted a histogram without a gradient. Where does the gradient come from?

Response: We accept this criticism. We have removed the word “gradient” and the “enrichment” framing here and throughout. Figure 1 is a stacked proportion chart, and we now describe it accurately as a cross-sectional comparison of proportions across WHO stage categories at a single point in time, not a within-patient trend (Results Section 3.4; Fig 1 legend). We report the proportions plainly (48.5% malignant at Stage I rising to 96.0% at Stage IV) without implying a longitudinal trajectory.

“The categorical analysis reinforced this gradient: 44/71 (62.0%) patients with pre-ART CD4 …” The statement implies something not supported; the figure had no gradient, and there is no longitudinal linkage between CD4 and tumour presence.

Response: We have rephrased this entirely (Results Section 3.4). We removed “reinforced this gradient” and the implication of a longitudinal CD4-to-tumour link. We now report only the cross-sectional categorical proportions (47/71, 66.2% of records with CD4 <200 were malignant vs 8/19, 42.1% of those with CD4 ≥500) and explicitly note these are cross-sectional, not longitudinal. (The exact figures also changed slightly with the corrected dataset.)

“Malignancy risk was heavily concentrated in the earliest ART period … This temporal gradient supports …” Inappropriate phrasing not supported by the data.

Response: Revised (Results Section 3.4; Fig 3 legend). We removed “temporal gradient” and the causal framing from the Results. We now report the cross-sectional proportions (87.1% of records diagnosed within 12 months of ART initiation were malignant vs 48.4% among those on ART >120 months) and state plainly that this is a comparison of proportions across categories, not evidence of a temporal or causal gradient. The two candidate mechanisms (IRIS unmasking and detection of occult malignancy at ART initiation) are now discussed as hypotheses in the Discussion rather than asserted in the Results.

“Although unsuppressed viraemia was numerically associated with a higher malignant proportion (16/22, 72.7% vs 70/134, 52.2%; OR 2.44 …) … Critically, the absolute majority of malignant tumours (70/86, 81.4%) occurred in virally suppressed patients …” The study did not study viral load as a continuous variable; the wording is not supported by the data (frequency). Refrain from “numerically associated.”

Response: We agree and have revised (Results Section 3.4). Viral load is described and analysed strictly as the dichotomous variable it was collected as (suppressed/unsuppressed); we never describe or model it as continuous. We removed “numerically associated.” The revised text simply states the observed proportions: a higher proportion of unsuppressed records were malignant than suppressed records (16/22, 72.7% vs 70/133, 52.6%), that this did not reach statistical significance (crude OR 2.39, 95% CI 0.82–7.92; FDR-adjusted p=0.141), and that most malignant tumours with viral load data occurred in virally suppressed records. (Denominators updated for the corrected dataset.)

Using “association” wording for crude and unadjusted estimates might confuse readers. Since no regression was conducted, refrain from using “association.”

Response: We have reviewed every use of “association” and “associated.” Where we retain the term, it now unambiguously refers to a bivariate statistical test result, and we state explicitly in Methods and in the Discussion that all estimates are crude, unadjusted, and hypothesis-generating, with no multivariable regression performed. We have removed causal or implied-mechanism phrasing from the Results throughout, confining interpretation to the Discussion.

KS-versus-other comparison: “KS patients were also significantly more likely to be male (41/65, 63.1% vs 32/73, 43.8%; p=0.024) and to have had advanced WHO stage at ART initiation (65.8% vs 29.2% …)…” See comment above.

Response: In addition to applying the same de-“gradient”/de-“association” language changes here, we identified and corrected a substantive error in the original Table 4: several rows had been computed on inconsistent denominators, and the KS-versus-non-KS comparison had inadvertently included benign tumours in the comparison group. In the revised manuscript, this comparison is correctly restricted to malignant tumours only (65 KS vs 73 other malignancies, n=138), and every row states its own denominator (Results Section 3.5; Table 4). The corrected advanced-WHO-stage figures are 47.3% (26/55) for KS versus 18.6% (13/70) for other malignancies (FDR-adjusted p=0.003); the corrected sex figures are 63.1% versus 43.8% male (FDR-adjusted p=0.041). We also note that Figures 4 and 5 have been regenerated to reflect this malignant-only restriction.

Temporal pattern of tumour registrations (“Annual tumour registrations peaked in 2018 … The pre-2020 peak should not be interpreted as a true epidemiological surge.”) This is not required.

Response: We have removed the standalone “Temporal pattern of tumour registrations” subsection from the Results. We retain only a brief factual note in the cohort-characteristics paragraph (Results Section 3.1) that most records were registered in 2017–2019 with a 2018 peak partly attributable to a data-quality programme, together with an explicit caution that this reflects registration/ascertainment rather than true incidence; this caution is necessary to prevent misreading and is now tied to a corresponding point in the Limitations.

F. Reviewer 1: Discussion

In the Discussion, it is not clear what the key findings are. The author notes that most people had Kaposi sarcoma but fails to highlight that screening and diagnosis for KS might have improved during this period. He alludes to well-established biological mechanisms but fails to discuss the results in the context of Mildmay or Uganda. The author needs to re-evaluate the results and produce a discussion tailored to his results that is clear and less redundant.

Response: We have substantially rewritten the Discussion (Section 4). The changes are:

1. The Discussion now opens by stating the key findings plainly and is organised around four clearly labelled findings drawn directly from our own results, rather than a longer list of general statements.

2. We now explicitly raise the possibility the reviewer identifies, that improved clinical recognition and coded documentation of KS at Mildmay over the study period (particularly around the 2018 data-quality programme) may have increased KS ascertainment, and we state honestly that we cannot fully separate a true rise in KS burden from improved detection and recording. This is now presented alongside, not instead of, the biological rationale, and is linked to the registration-pattern caution in the Results and Limitations.

3. We have reduced redundancy: the mechanistic HHV-8/immune-reconstitution material is now more concise and is explicitly connected to our cohort’s findings (for example, the KS-versus-other-malignancies contrast in Table 4, showing KS patients had a median of only 4 months on ART at diagnosis and higher WHO stage), rather than being presented as general background.

4. We have grounded the interpretation in the Mildmay/Uganda context, including the implications for this centre’s early-ART follow-up screening, the Uganda-specific cervical cancer prevention context, and the site-level research agenda that follows from these specific results.

5. We reiterate throughout the Discussion that all findings are cross-sectional and hypothesis-generating.

G. Additional changes made at revision

Beyond the specific points above, and prompted by the requirement to re-run the analysis, we:

• Excluded one tumour record whose WHO clinical stage was coded as an uninterpretable “unknown” value outside the four-stage WHO scale; the analytic dataset is now 219 records from 216 patients (Methods Section 2.2). All descriptive and inferential results were regenerated accordingly, so some numbers differ slightly from the original submission.

• Added Benjamini–Hochberg FDR-adjusted p-values throughout (Tables 3 and 4).

• Added a patient-level sensitivity analysis (S2 Table).

• Corrected the Table 4 denominators and the corresponding Figures 4 and 5 to compare KS against other malignancies only.

• Verified all reference details.

We are grateful to the reviewer and editor for comments that have materially strengthened the manuscript, and we hope the revised version is now suitable for publication in PLOS ONE.

Yours sincerely,

Dr. Joseph Mwaka, on behalf of all authors Mildmay Research Centre, Kampala, Uganda

Attachment Submitted filename: Response_to_Reviewers.docx

# Editor report: Decision Letter 1

**DOI:** 10.1371/journal.pone.0358066.r003

**Authors:** Felix Bongomin

26 Aug 2026

Spectrum and Immunovirological Determinants of Tumours Among People Living with HIV in Uganda: A Retrospective Cross sectional Study from a Specialised HIV Centre, 2017–2026

PONE-D-26-21762R1

Dear Dr. Mwaka,

We’re pleased to inform you that your manuscript has been judged scientifically suitable for publication and will be formally accepted for publication once it meets all outstanding technical requirements.

Within one week, you’ll receive an e-mail detailing the required amendments. When these have been addressed, you’ll receive a formal acceptance letter and your manuscript will be scheduled for publication.

An invoice will be generated when your article is formally accepted. Please note, if your institution has a publishing partnership with PLOS and your article meets the relevant criteria, all or part of your publication costs will be covered. Please make sure your user information is up-to-date by logging into Editorial Manager at Editorial Manager® and clicking the ‘Update My Information' link at the top of the page. For questions related to billing, please contact billing support.

If your institution or institutions have a press office, please notify them about your upcoming paper to help maximize its impact. If they’ll be preparing press materials, please inform our press team as soon as possible -- no later than 48 hours after receiving the formal acceptance. Your manuscript will remain under strict press embargo until 2 pm Eastern Time on the date of publication. For more information, please contact onepress@plos.org.

Kind regards,

Felix Bongomin, MB ChB, Ph.D., FECMM

Academic Editor

PLOS One

Additional Editor Comments (optional):

Reviewers' comments:

# Editor report: Acceptance letter

**DOI:** 10.1371/journal.pone.0358066.r004

**Authors:** Felix Bongomin

PONE-D-26-21762R1

PLOS One

Dear Dr. Mwaka,

I'm pleased to inform you that your manuscript has been deemed suitable for publication in PLOS One. Congratulations! Your manuscript is now being handed over to our production team.

At this stage, our production department will prepare your paper for publication. This includes ensuring the following:

* All references, tables, and figures are properly cited

* All relevant supporting information is included in the manuscript submission,

* There are no issues that prevent the paper from being properly typeset

You will receive further instructions from the production team, including instructions on how to review your proof when it is ready. Please keep in mind that we are working through a large volume of accepted articles, so please give us a few days to review your paper and let you know the next and final steps.

Lastly, if your institution or institutions have a press office, please let them know about your upcoming paper now to help maximize its impact. If they'll be preparing press materials, please inform our press team within the next 48 hours. Your manuscript will remain under strict press embargo until 2 pm Eastern Time on the date of publication. For more information, please contact onepress@plos.org.

You will receive an invoice from PLOS for your publication fee after your manuscript has reached the completed accept phase. If you receive an email requesting payment before acceptance or for any other service, this may be a phishing scheme. Learn how to identify phishing emails and protect your accounts at https://explore.plos.org/phishing.

If we can help with anything else, please email us at customercare@plos.org.

Thank you for submitting your work to PLOS One and supporting open access.

Kind regards,

PLOS One Editorial Office Staff

on behalf of

Dr. Felix Bongomin

Academic Editor

PLOS One
